# Supplementary material for: RNA-coupled CRISPR Screens Reveal ZNF207 as a Regulator of LMNA Aberrant Splicing in Progeria
Source: bioRxiv. 2025 Apr 26:2025.04.25.648738. Preprint. [Version 1] doi: 10.1101/2025.04.25.648738 (PMC12191140; doi:10.1101/2025.04.25.648738)
Supplement: 1 [file NIHPP2025.04.25.648738V1-supplement-1.pdf]

bioRxiv preprint doi: https://doi.org/10.1101/2025.04.25.648738; this version posted April 27, 2025. The copyright holder for this preprint (which was not certified by peer review) is the author/funder. This article is a US Government work. It is not subject to copyright under 17 USC 105 and is also made available for use under a CC0 license.

Supplementary Figure 1

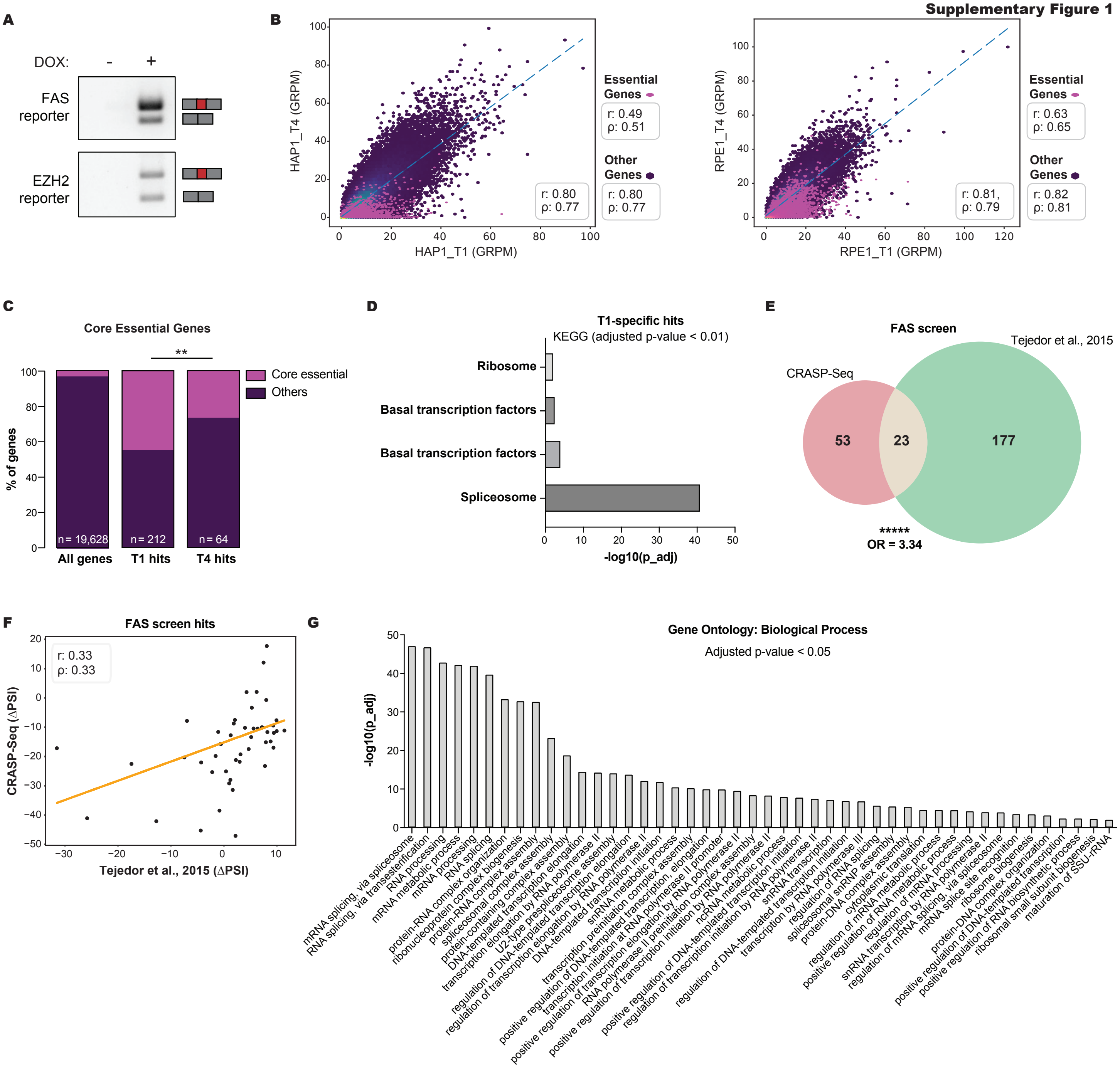

Supplementary Figure 1: CRASP-Seq effectively identifies splicing regulatory factors. Related to Figure 1.

(A) RT-PCR analysis of FAS and EZH2 minigene reporters expressed in HAP1 cells transduced with CRASP-Seq vectors. Cells were treated with 1 µg/mL doxycycline (+) or not (–) for 20 hours before RNA extraction.

(B) Correlation analysis of guide read counts per million (GRPM) at early (T1) and late (T4) time points in the FAS CRASP-Seq screen. Scatter plots and Pearson correlation coefficients are shown for HAP1 (left) and RPE1 (right) cell lines, with separate analysis of common essential genes versus other genes. "r" denotes the Pearson correlation coefficient and "ρ" represents the Spearman's rank correlation coefficient.

(C) Bar plots showing the percentage of core-essential genes[94] among all library-targeted genes, T1 CRASP-Seq hits, and T4 CRASP-Seq hits. **p-value = 0.0092; odds ratio = 2.25; Fisher's exact test.

(D) Enrichment of Kyoto Encyclopedia of Genes and Genomes (KEGG) biological pathways among T1-specific (i.e., lost in the T4 point) screen hits. Pathways with adjusted p-value < 0.01 are displayed.

(E) Venn diagram comparing FAS screen hits identified by CRASP-Seq with those from a genome-wide siRNA robotics screen.[15] ****p-value < 0.00001; OR = 3.34; Fisher's exact test.

(F) Correlation plot of differential splicing (ΔPSI) values for gene hits identified by CRASP-Seq. ΔPSI values are shown for CRASP-Seq versus the siRNA robotics screen.[15] "r" denotes the Pearson correlation coefficient and "ρ" represents the Spearman's rank correlation coefficient.

(G) Gene Ontology (GO) analysis of biological processes enriched among regulators of FAS and EZH2 splicing events identified via the CRASP-Seq pipeline (related to Figure 1D). Hits with adjusted p-value < 0.05 are displayed.

bioRxiv preprint doi: https://doi.org/10.1101/2025.04.25.648738; this version posted April 27, 2025. The copyright holder for this preprint (which was not certified by peer review) is the author/funder. This article is a US Government work. It is not subject to copyright under 17 USC 105 and is also made available for use under a CC0 license.

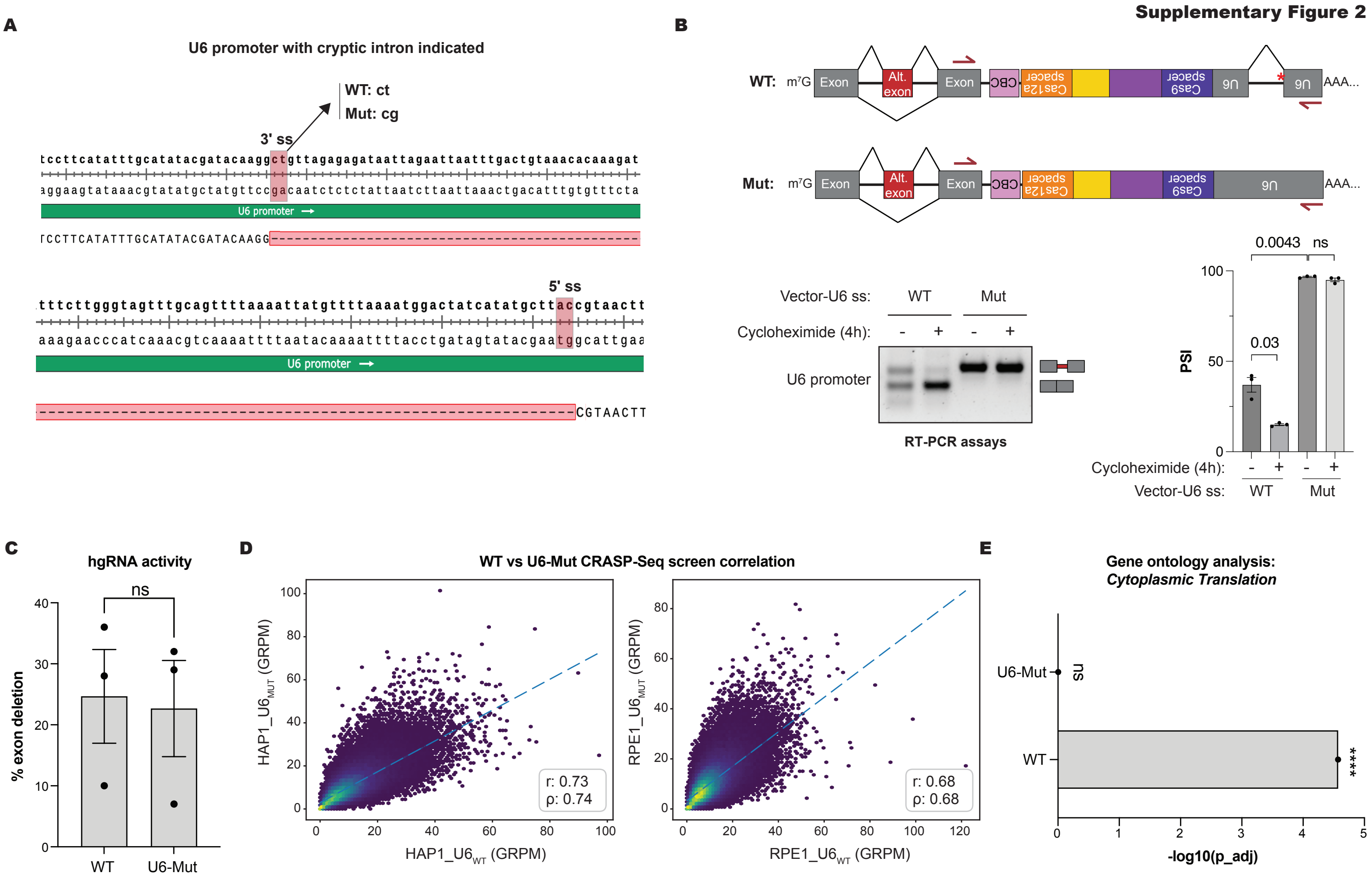

Supplementary Figure 2: A point mutation in the CRASP-Seq vector eliminates a cryptic 3′ splice site and prevents spurious NMD. Related to Figure 1.

(A) Sequence of the U6 promoter region showing the cryptic 3' and 5' splice sites and the associated intron. The position of the introduced point mutation designed to inactivate the cryptic splice site is highlighted. WT: wild-type; Mut: U6 point mutation.

(B) RT-PCR analysis (bottom left) and PSI quantification (bottom right) for RNA extracted from HAP1 cells transduced with wild-type (WT) or U6 point-mutated (Mut) CRASP-Seq vectors. Cells were treated with 100 µg/mL cycloheximide or DMSO for 4 hours prior to RNA extraction. A schematic illustrating the primer annealing sites is shown at the top. Data from independent replicate experiments are shown. Statistical significance was assessed using a two-tailed Welch's t-test.

(C) Editing efficiency of wild-type (WT) and U6 point-mutated (Mut) CRASP-Seq vectors was evaluated using HPRT1 exon deletion as a readout.[33] Three independent HPRT1 exon deletion hgRNAs were transduced, and deletion efficiency was measured from PCR-amplified genomic DNA. Statistical significance was assessed using a two-way paired t-test.

(D) Correlation of guide read counts per million (GRPM) between CRASP-Seq screens conducted with wild-type (WT) and U6 point-mutated (U6-Mut) vectors. Scatter plots and Pearson correlation coefficients are shown for HAP1 (left) and RPE1 (right) cell lines. "r" denotes the Pearson correlation coefficient and "ρ" represents the Spearman's rank correlation coefficient.

(E) Bar plot showing the –log10 transformed adjusted p-values for enrichment of cytoplasmic translation-related terms among CRASP-Seq screen hits identified using wild-type (WT) versus U6 point-mutated (U6-Mut) lentiviral libraries.

bioRxiv preprint doi: https://doi.org/10.1101/2025.04.25.648738; this version posted April 27, 2025. The copyright holder for this preprint (which was not certified by peer review) is the author/funder. This article is a US Government work. It is not subject to copyright under 17 USC 105 and is also made available for use under a CC0 license.

Supplementary Figure 3

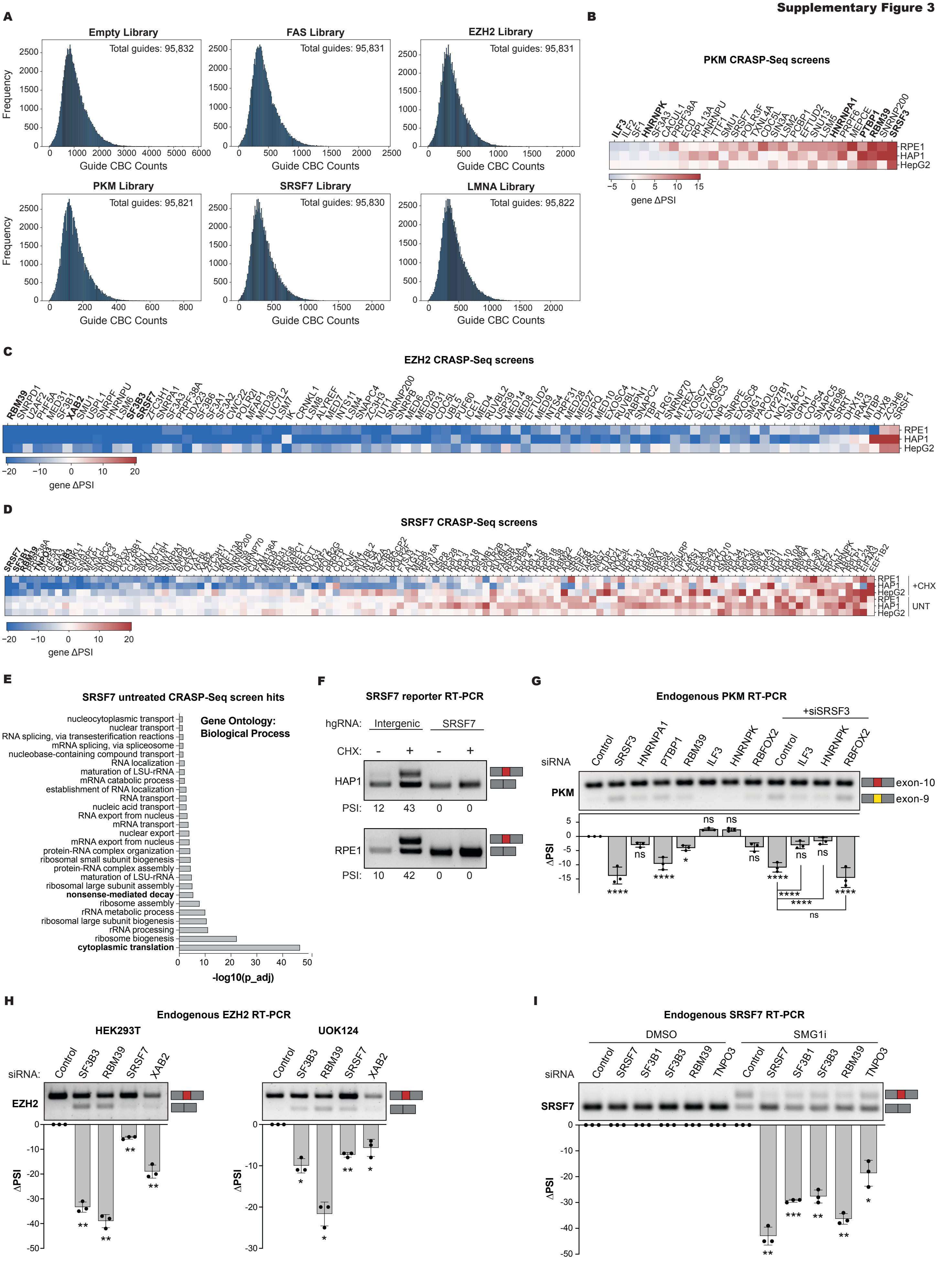

bioRxiv preprint doi: https://doi.org/10.1101/2025.04.25.648738; this version posted April 27, 2025. The copyright holder for this preprint (which was not certified by peer review) is the author/funder. This article is a US Government work. It is not subject to copyright under 17 USC 105 and is also made available for use under a CC0 license.

Supplementary Figure 3: Genome-wide CRASP-Seq screens identify regulators of disease-related splicing events. Related to Figure 2.
(A) Histogram showing the distribution of read counts for the 95,893 hgRNAs in the CRASP-Seq library. Separate histograms are displayed for the empty library as well as all five reporter libraries.
(B) Heatmap of genes identified by CRASP-Seq screens as regulators of *PKM* alternative splicing. Differential splicing levels (ΔPSI) relative to intergenic controls are shown. Genes promoting exon-10 inclusion (PKM2 isoform) with positive ΔPSI values are highlighted in red, while genes promoting exon-9 inclusion (PKM1 isoform) are highlighted in blue. Genes selected for RT-PCR validation are highlighted in bold.
(C) Heatmap of genes identified as regulators of EZH2 exon-14 alternative splicing in CRASP-Seq screens. Differential splicing levels (ΔPSI) relative to intergenic controls are shown, with genes promoting exon-14 inclusion highlighted in blue. Genes selected for RT-PCR validation are highlighted in bold.
(D) Heatmap of genes identified as regulators of SRSF7 poison exon splicing in CRASP-Seq screens. Differential splicing levels (ΔPSI) relative to intergenic controls are shown both with and without cycloheximide treatment. Genes promoting poison exon inclusion are highlighted in blue. Genes selected for RT-PCR validation are highlighted in bold.
(E) Gene Ontology (GO) analysis of biological processes enriched among regulators of the *SRSF7* poison exon identified via the CRASP-Seq pipeline in untreated cells. Hits with adjusted p-value < 0.05 are displayed.
(F) RT-PCR validation of alternative splicing for SRSF7 poison exon using the CRASP-Seq *SRSF7* minigene reporter in HAP1 (top) and RPE1 (bottom) cells co-expressing intergenic or SRSF7-targeting hgRNAs. Cells were treated with 100 μg/mL cycloheximide or DMSO for 4 hours prior to RNA extraction.
(G) RT-PCR analysis of endogenous PKM alternative splicing in HEK293 cells. The indicated genes (or combinations) were knocked down using siRNAs for 50 hours before RNA extraction. Quantification of ΔPSI values from three independent experiments are displayed below the gel. Data are represented as mean ± standard deviation (SD). *p-value < 0.05, ****p-value < 0.0001; two-way ANOVA.
(H) RT-PCR analysis of endogenous EZH2 alternative splicing in HEK293 (left) and UOK124 renal cell carcinoma (right) cell lines. The indicated genes were knocked down using siRNA for 48 hours before RNA extraction. Quantification of ΔPSI values from three independent experiments are displayed below the gel. Data are presented as mean ± SD. *p-value < 0.05, **p-value < 0.01; Welch's t-test with Holm-Šídák method multiple correction testing.
(I) RT-PCR analysis of endogenous SRSF7 poison exon splicing in HEK293 cells. Cells were treated with 0.5 μM SMG1i or DMSO for 8 hours prior to RNA extraction. Quantifications of ΔPSI values from three independent experiments are displayed below the gel. Data are presented as mean ± SD. *p-value < 0.05, **p-value < 0.01, ***p-value < 0.001; Welch's t-test with Holm-Šídák method multiple correction testing.

bioRxiv preprint doi: https://doi.org/10.1101/2025.04.25.648738; this version posted April 27, 2025. The copyright holder for this preprint (which was not certified by peer review) is the author/funder. This article is a US Government work. It is not subject to copyright under 17 USC 105 and is also made available for use under a CC0 license.

Supplementary Figure 4

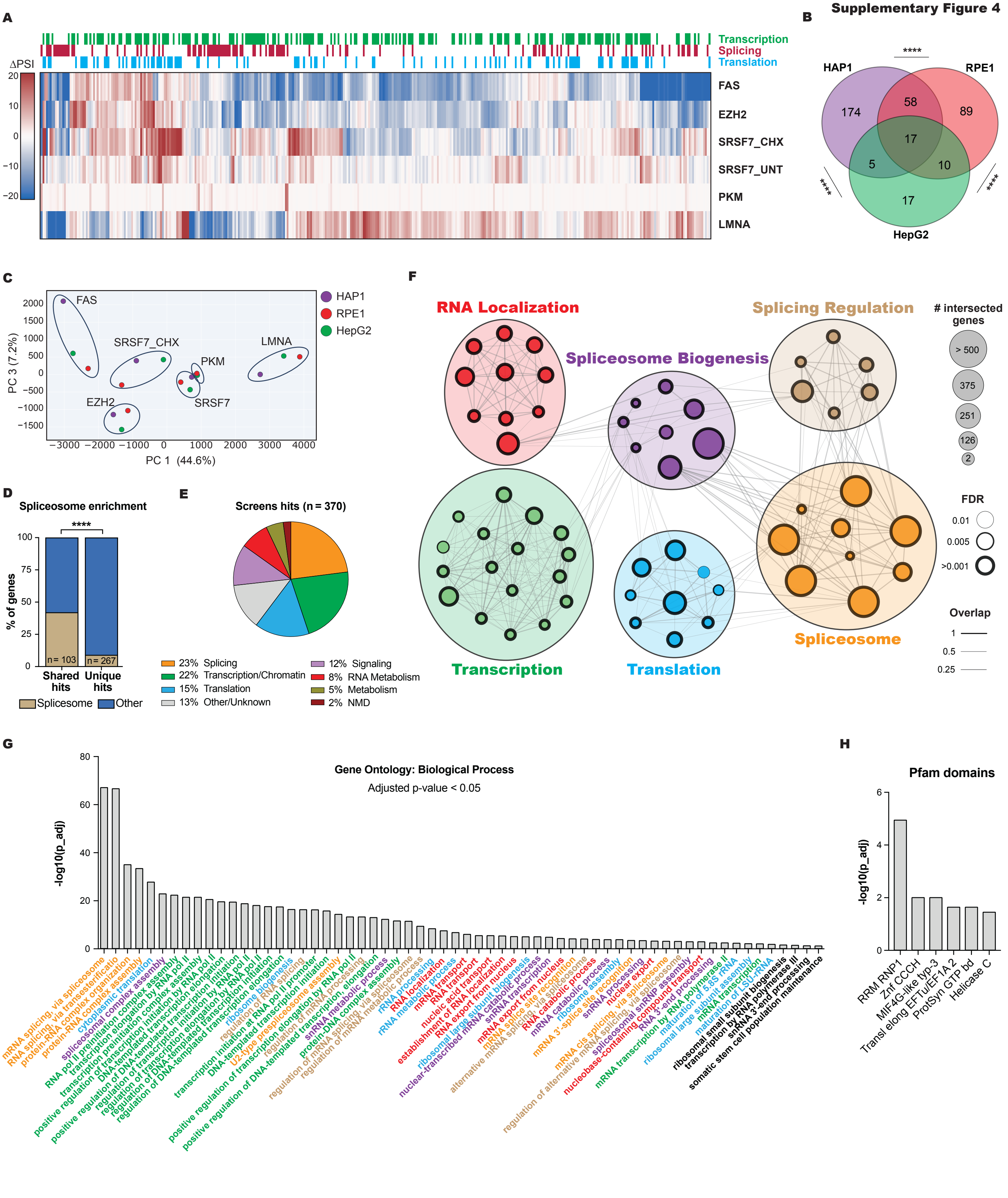

bioRxiv preprint doi: https://doi.org/10.1101/2025.04.25.648738; this version posted April 27, 2025. The copyright holder for this preprint (which was not certified by peer review) is the author/funder. This article is a US Government work. It is not subject to copyright under 17 USC 105 and is also made available for use under a CC0 license.

Supplementary Figure 4: Genome-wide identification and functional characterization of alternative splicing regulators. Related to Figure 2.

(A) Heatmap of ΔPSI values for CRASP-Seq screen hits across the five alternative splicing events in HepG2 cells. Functional annotations of genes are shown above the heatmap.

(B) Venn diagram showing the overlap of CRASP-Seq screen hits identified across all five splicing reporters in three different cell lines. ****p-value < 0.0001; Fisher's exact test.

(C) Principal component analysis (PCA) of the six CRASP-Seq screens across the three different cell lines.

(D) Bar plots depicting the percentage of core spliceosome genes (as defined by containing CORUM spliceosome terms) among shared hits identified across two or more reporters compared to unique hits identified by only one reporter. p-value < 0.0001; odds ratio = 7.32; Fisher's exact test.

(E) Pie chart depicting the functional classification of genes identified as hits in the genome-wide CRASP-Seq screens. Categories were curated through literature review and functional enrichment analysis using the g:Profiler Gene Ontology (GO) database.

(F) GO enrichment analysis of regulators identified by all CRASP-Seq regulatory screens. Only significantly enriched biological processes with adjusted p-value < 0.01 are displayed.

(G) Detailed GO enrichment analysis of biological processes associated with regulators of all reporters identified via the CRASP-Seq pipeline. Hits with adjusted p-value < 0.05 are shown. GO terms are color-coded to align with the functional categories shown in Supplementary Figure 4F.

(H) Pfam domain enrichment analysis of regulators identified by all CRASP-Seq regulatory screens using Enrichr.[95] Only significantly enriched domains with adjusted p-value < 0.05 are displayed.

bioRxiv preprint doi: https://doi.org/10.1101/2025.04.25.648738; this version posted April 27, 2025. The copyright holder for this preprint (which was not certified by peer review) is the author/funder. This article is a US Government work. It is not subject to copyright under 17 USC 105 and is also made available for use under a CC0 license.

Supplementary Figure 5

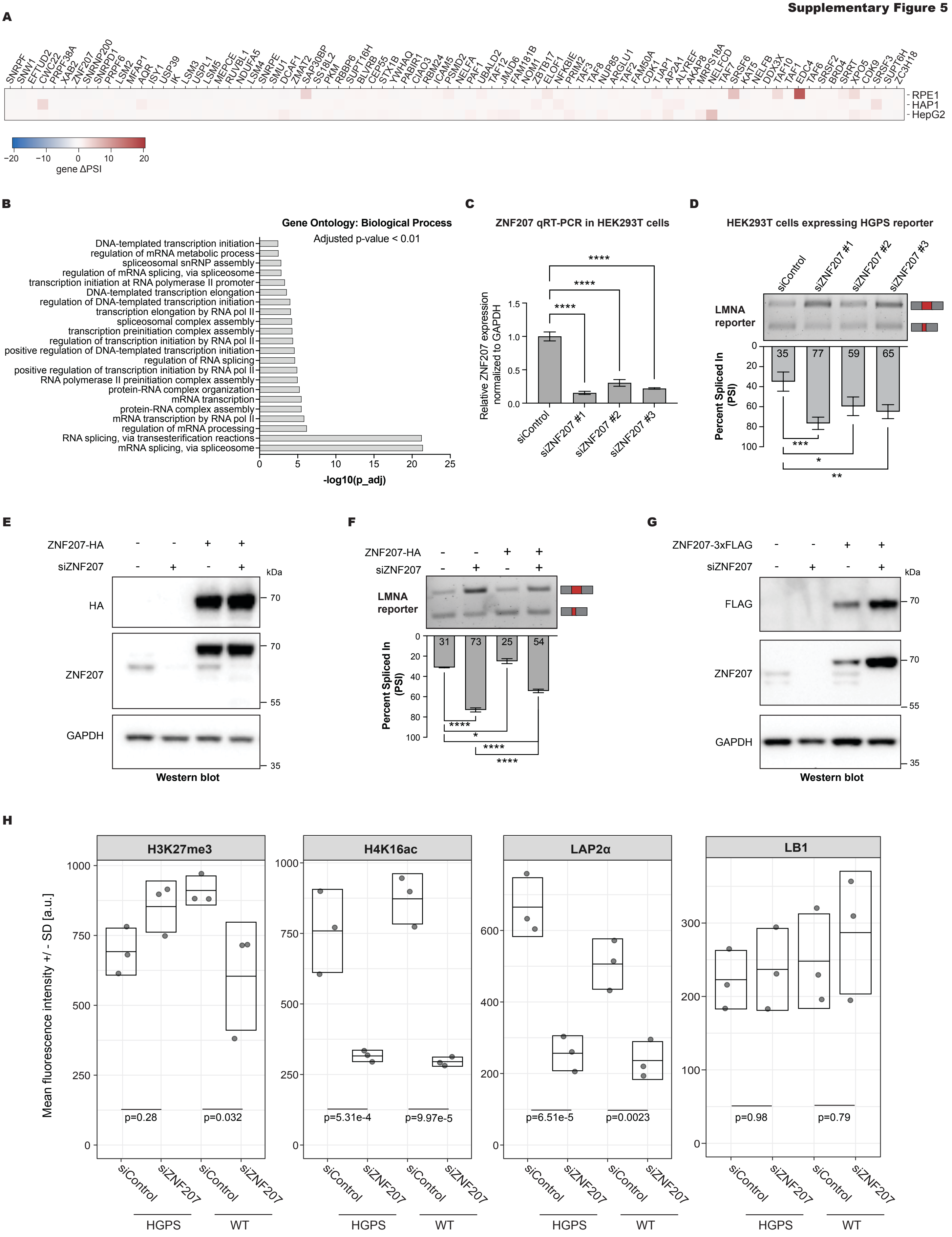

bioRxiv preprint doi: https://doi.org/10.1101/2025.04.25.648738; this version posted April 27, 2025. The copyright holder for this preprint (which was not certified by peer review) is the author/funder. This article is a US Government work. It is not subject to copyright under 17 USC 105 and is also made available for use under a CC0 license.

Supplementary Figure 5: ZNF207 is a positive regulator of progerin aberrant splicing. Related to Figure 3.

(A) Heatmap showing ΔPSI values for wild-type *LMNA* CRASP-Seq screens for the hits identified in the mutant *LMNA* screens across HAP1, RPE1, and HepG2 cell lines.

(B) Gene Ontology (GO) enrichment analysis of biological processes among *LMNA* CRASP-Seq screen hits with adjusted p-value < 0.01.

(C) Real-time quantitative RT-PCR measuring ZNF207 transcript depletion in HEK293 cells transfected with three independent siRNAs. Quantification of transcript levels from three independent experiments are displayed. Data are presented as mean ± SD. ****p-value < 0.0001; Dunnett's multiple comparisons test following one-way ANOVA.

(D) RT-PCR analysis of aberrant *LMNA* splicing in RNA from HEK293T cells transduced with the mutant *LMNA* minigene reporter and treated with three independent siRNAs targeting ZNF207. Quantification of PSI values from three independent experiments are shown below the gel. Data are presented as mean ± SD. *p-value < 0.05, **p-value < 0.01, ***p-value < 0.001; Dunnett's multiple comparisons test following one-way ANOVA.

(E) Western blot analysis of ZNF207 in HEK293T cells expressing the *LMNA* minigene reporter, transiently transfected with either a siRNA-resistant 3×HA C-terminal-tagged ZNF207 ORF or an empty vector. Cells were treated with either non-targeting siRNA control or siRNA targeting endogenous ZNF207 (siZNF207). Blots were probed with antibodies against ZNF207, HA tag, and GAPDH (loading control).

(F) RT-PCR analysis of *LMNA* splicing in HEK293 cells expressing the *LMNA* minigene reporter and treated with ZNF207-targeting siRNA and/or ectopically expressing an siRNA-resistant ZNF207 ORF. Quantification of PSI values from three independent experiments are shown below the gel. Data are presented as mean ± SD. **p-value < 0.01, ****p-value < 0.0001; Dunnett's multiple comparisons test following one-way ANOVA.

(G) Western blot analysis of ZNF207 in HGPS patient-derived immortalized fibroblasts stably transduced with either a siRNA-resistant 3×FLAG C-terminal-tagged ZNF207 ORF or an empty vector. Cells were treated with either non-targeting siRNA control or siRNA targeting endogenous ZNF207 (siZNF207). Blots were probed with antibodies against ZNF207, FLAG, and GAPDH (loading control).

(H) Single cell high throughput immunofluorescence quantification of LAP2α, H3K27me3, H4K16ac and LB1 expression levels in immortalized human fibroblasts treated with siZNF207 or siCTL for 15 days. At least 600 cells were analyzed in each experiment and represented as the distribution of average mean fluorescence intensity. The values from three independent experiments ± SD are plotted (see Methods). Statistical differences were analyzed by one-way ANOVA followed by the Dunnett's test using the WT siCTL cell line as the negative control for WT siZNF207 and the HGPS siCTL as the negative control for HGPS siZNF207.

bioRxiv preprint doi: https://doi.org/10.1101/2025.04.25.648738; this version posted April 27, 2025. The copyright holder for this preprint (which was not certified by peer review) is the author/funder. This article is a US Government work. It is not subject to copyright under 17 USC 105 and is also made available for use under a CC0 license.

Supplementary Figure 6

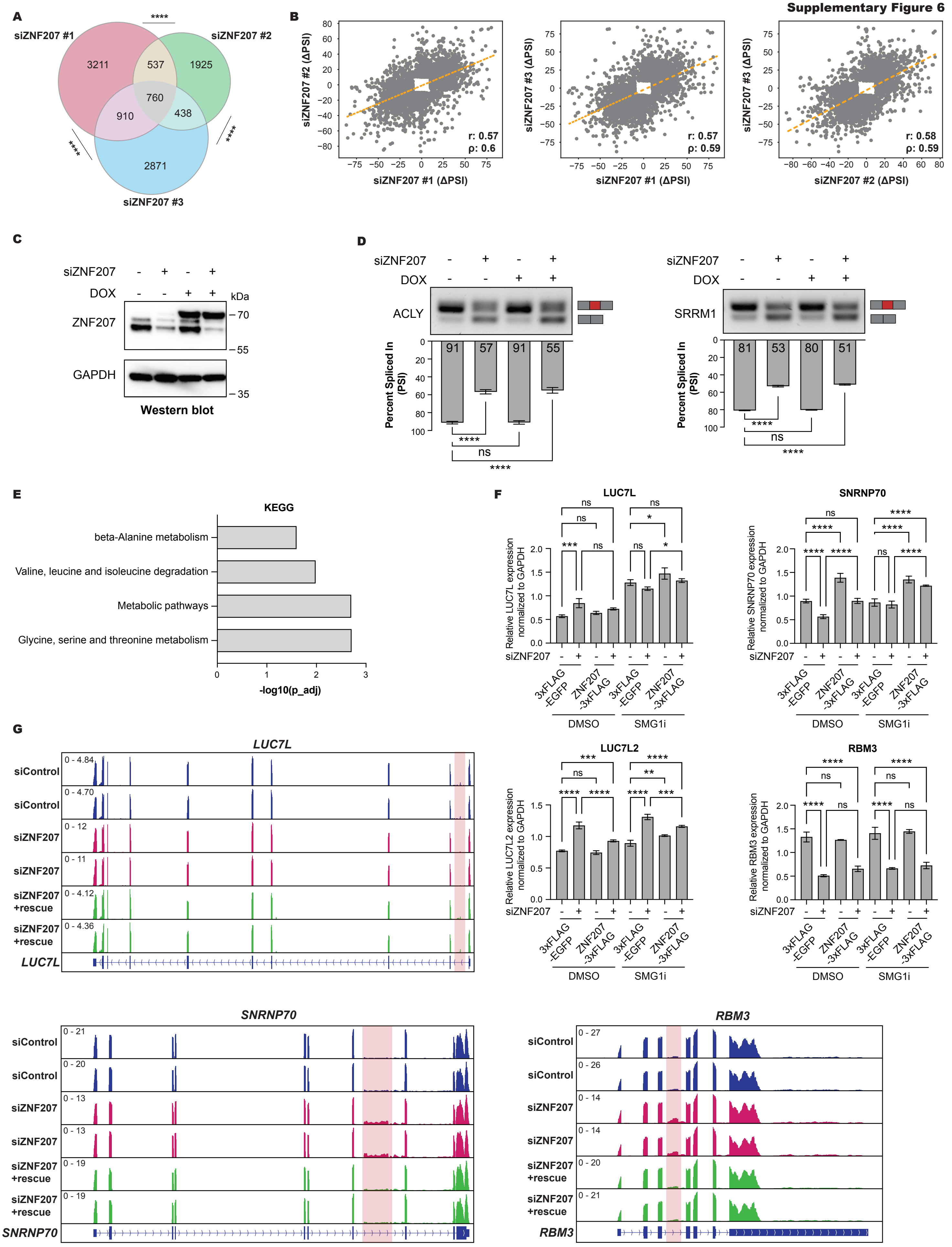

bioRxiv preprint doi: https://doi.org/10.1101/2025.04.25.648738; this version posted April 27, 2025. The copyright holder for this preprint (which was not certified by peer review) is the author/funder. This article is a US Government work. It is not subject to copyright under 17 USC 105 and is also made available for use under a CC0 license.

Supplementary Figure 6: ZNF207 is an alternative splicing regulator. Related to Figure 4.

(A) Venn diagram illustrating the overlap of regulated splicing events ($|\Delta PSI| \geq 10$ and probability ≥ 0.95) across the three independent siRNA sequences targeting ZNF207. ****p-value < 0.0001; Fisher's exact test.

(B) Correlation of PSI changes (ΔPSI) in HEK293T cells transfected with three independent siRNA sequences targeting ZNF207. Significant events for each siRNA treatment are highlighted using distinct colors. "r" denotes the Pearson correlation coefficient and "ρ" represents the Spearman's rank correlation coefficient.

(C) Western blot analysis of ZNF207 in HEK293 Flp-In cells expressing doxycycline-inducible, siRNA-resistant 3×FLAG N-terminal tagged ZNF207 ORF. Cells were treated with non-targeting siRNA control or siRNA targeting endogenous ZNF207 (siZNF207). Blots were probed with antibodies against ZNF207, and GAPDH (loading control).

(D) RT-PCR analysis of alternative splicing for *ACLY* (left) and *SRRM1* (right) in HEK293 Flp-In cells treated with ZNF207-targeting siRNA and/or expressing siRNA-resistant N-terminally tagged ZNF207 ORF. Quantification of PSI values from three independent experiments are shown below the gel. Data are presented as mean ± SD. ****p < 0.0001; Dunnett's multiple comparisons test following one-way ANOVA.

(E) Enrichment of KEGG biological pathways among genes showing differential expression after ZNF207 depletion, which are rescued by ZNF207 reintroduction (as shown in Figure 4E). Only terms with adjusted p-value < 0.05 are shown.

(F) Real-time quantitative RT-PCR analysis of transcript levels for *LUC7L*, *LUC7L2*, *RBM3*, and *SNRNP70* in HEK293 Flp-In cells expressing a doxycycline-inducible, siRNA-resistant 3×FLAG C-terminal tagged ZNF207 ORF. Cells were treated with either non-targeting siRNA controlor siRNA targeting endogenous ZNF207 (siZNF207) and further treated with either 0.5 μM SMG1 NMD inhibitor (SMG1i) or DMSO for 6 hours prior to RNA extraction. Quantification of transcript levels from three independent experiments are displayed. Data are presented as mean ± SD. ****p-value < 0.0001, ***p-value < 0.001, **p-value < 0.01, *p-value < 0.05; Šídák's multiple comparisons testing following one-way ANOVA.

(G) Genome browser tracks highlighting alternative splicing events in *LUC7L*, *SNRNP70*, and *RBM3*, genes whose expression is regulated by ZNF207. Tracks represent data from two independent RNA-Seq replicates under three conditions: cells treated with control siRNAs (siControl), siRNAs targeting ZNF207 (siZNF207), and siZNF207-treated cells rescued by induced ZNF207 expression. Normalized reads per million (RPM) are displayed on the left of each track. The regulated splicing events are highlighted.

bioRxiv preprint doi: https://doi.org/10.1101/2025.04.25.648738; this version posted April 27, 2025. The copyright holder for this preprint (which was not certified by peer review) is the author/funder. This article is a US Government work. It is not subject to copyright under 17 USC 105 and is also made available for use under a CC0 license.

Supplementary Figure 7

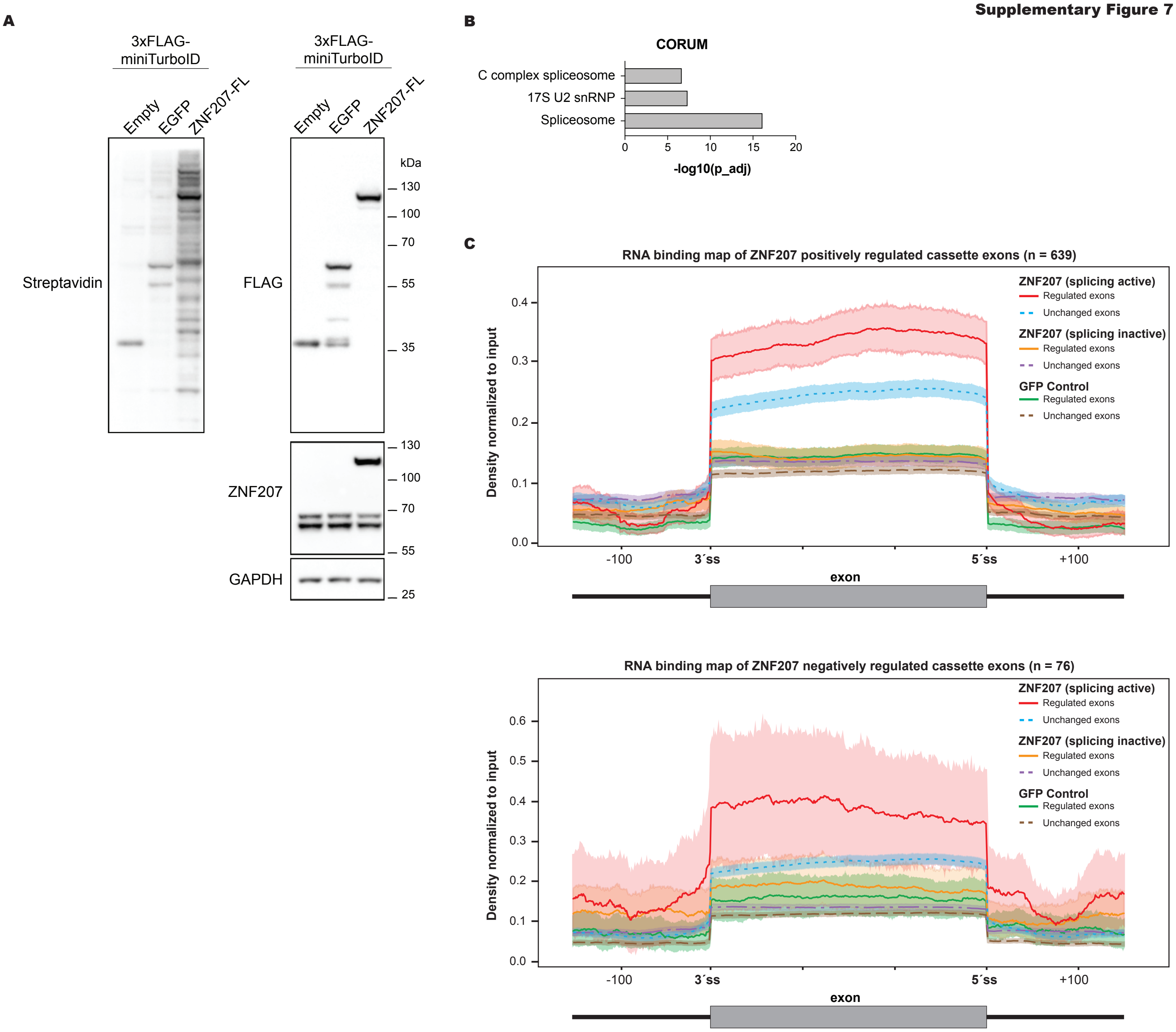

Supplementary Figure 7: ZNF207 directly impacts alternative splicing. Related to Figure 5.

(A) Western blot analysis of HEK293 Flp-In cells expressing 3×FLAG-miniTurboID-tagged constructs, including an empty vector, EGFP, or ZNF207 (C-terminal tagged with 3×FLAG-miniTurboID). The blot was probed with streptavidin (for biotinylation detection), ZNF207, FLAG, and GAPDH antibodies.

(B) CORUM complex enrichment analysis of ZNF207 proximal interactors identified via TurboID experiments. All enriched terms have adjusted p-value < 0.0001.

(C) Average eCLIP signal profiles for C-terminal FLAG-tagged ZNF207 over exons positively (top; n = 639) or negatively (bottom; n = 76)) regulated by ZNF207 and over unchanged alternative exons (n = 3,745). Signal profiles are compared to the negative control GFP and N-terminal FLAG-tagged ZNF207 (lacking splicing activity) for the same exon subsets.

bioRxiv preprint doi: https://doi.org/10.1101/2025.04.25.648738; this version posted April 27, 2025. The copyright holder for this preprint (which was not certified by peer review) is the author/funder. This article is a US Government work. It is not subject to copyright under 17 USC 105 and is also made available for use under a CC0 license.

Supplementary Figure 8

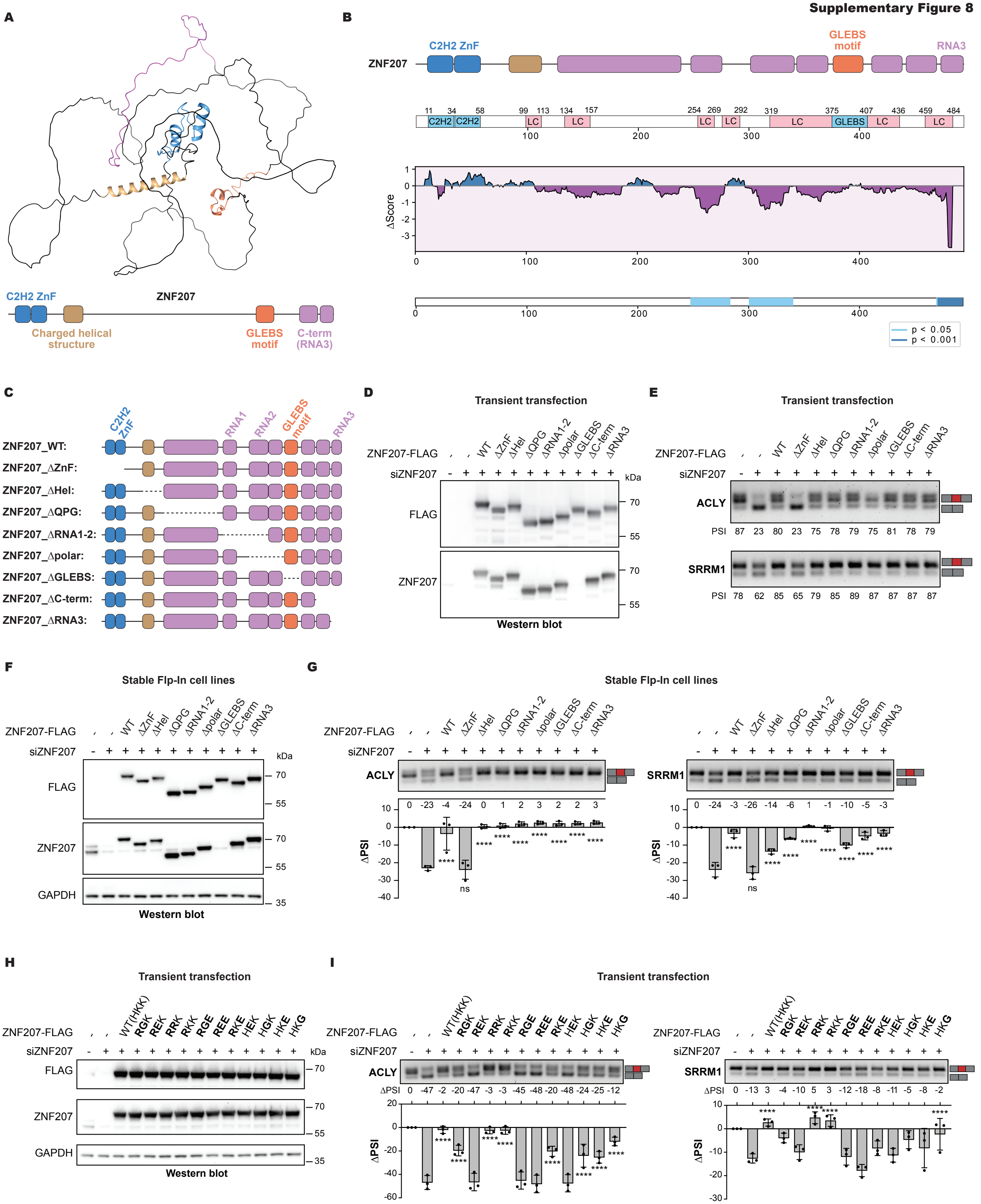

bioRxiv preprint doi: https://doi.org/10.1101/2025.04.25.648738; this version posted April 27, 2025. The copyright holder for this preprint (which was not certified by peer review) is the author/funder. This article is a US Government work. It is not subject to copyright under 17 USC 105 and is also made available for use under a CC0 license.

Supplementary Figure 8: Zinc finger domains are essential for ZNF207 splicing regulatory activity. Related to Figure 6.
(A) AlphaFold prediction of the full-length ZNF207 protein. Key structural regions are highlighted: the C2H2 zinc finger domain in blue, the helical structure in brown, the GLEBS motif in orange, and the C-terminal regions, predicted to be disordered and RNA-binding, in pink. Corresponding features are similarly annotated in the schematic below.
(B) HydRA analysis predicting RNA-binding regions within ZNF207. Occlusion maps illustrate regions with differential RNA-binding activity: purple regions represent Δscore < 0, while blue regions represent Δscore > 0. Statistically significant regions ($p < 0.05$ or $p < 0.001$) are highlighted in light blue and dark blue, respectively, on the bottom track. The coordinates of protein domains and annotated regions are displayed at the top.
(C) Schematic representation of ZNF207 truncation mutants used in the figure.
(D) Western blot analysis of ZNF207 truncation mutants transiently transfected into HEK293T cells. Cells were treated with control siRNA (siControl) or siRNA targeting endogenous ZNF207 (siZNF207). Blots were probed with antibodies against ZNF207, and FLAG.
(E) RT-PCR analysis of ACLY (top) and SRRM1 (bottom) alternative splicing in HEK293 cells treated with ZNF207-targeting siRNA and transiently transfected with siRNA-resistant, C-terminally 3×FLAG-tagged ZNF207 truncation mutants. Quantification of PSI values are shown below the gel.
(F) Western blot analysis of siRNA-resistant 3×FLAG C-terminal tagged ZNF207 truncation mutants expressed in HEK293 Flp-In cells under doxycycline induction. Cells were treated with non-targeting siRNA control or siRNA targeting endogenous ZNF207 (siZNF207). Western blots were probed with antibodies against ZNF207, FLAG (to detect tagged constructs), and GAPDH (loading control).
(G) RT-PCR analysis of ACLY (left) and SRRM1 (right) alternative splicing in HEK293 Flp-In cells treated with ZNF207-targeting siRNA and/or expressing siRNA-resistant wild-type or truncation mutant ZNF207 ORFs. Quantification of ΔPSI values from three independent experiments are shown below the gel. Data are presented as mean ± SD. Statistical comparisons were performed relative to the siZNF207 sample without ZNF207 ORF expression. ****$p < 0.0001$; Dunnett's multiple comparisons test following one-way ANOVA.
(H) Western blot analysis of ZNF207 mutants at residues H41, K42, and K43 (wild-type: HKK), transiently expressed in HEK293T cells. Cells were transfected with either control siRNA (siControl) or siRNA targeting endogenous ZNF207 (siZNF207). Blots were probed with antibodies against ZNF207, FLAG, and GAPDH (loading control).
(I) RT-PCR analysis of ACLY (left) and SRRM1 (right) alternative splicing in HEK293 cells treated with ZNF207-targeting siRNA and transiently transfected with siRNA-resistant, C-terminally 3×FLAG-tagged ZNF207 mutants. Quantification of ΔPSI values from three independent experiments are shown below the gel. Data are presented as mean ± SD. Statistical comparisons were performed relative to the siZNF207 sample without ZNF207 ORF expression. ****$p < 0.0001$; Dunnett's multiple comparisons test following one-way ANOVA.

bioRxiv preprint doi: https://doi.org/10.1101/2025.04.25.648738; this version posted April 27, 2025. The copyright holder for this preprint (which was not certified by peer review) is the author/funder. This article is a US Government work. It is not subject to copyright under 17 USC 105 and is also made available for use under a CC0 license.

Supplementary Figure 9

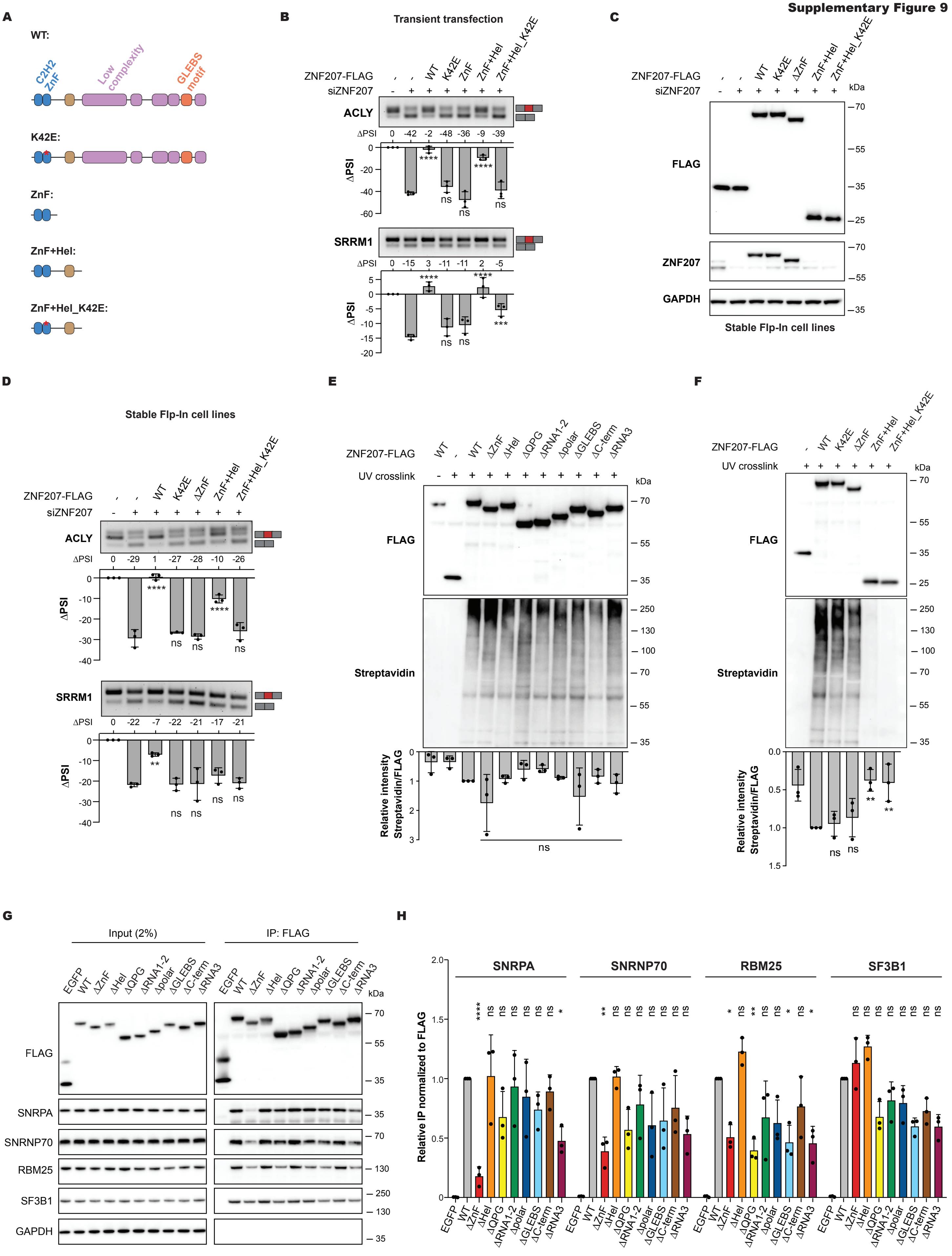

bioRxiv preprint doi: https://doi.org/10.1101/2025.04.25.648738; this version posted April 27, 2025. The copyright holder for this preprint (which was not certified by peer review) is the author/funder. This article is a US Government work. It is not subject to copyright under 17 USC 105 and is also made available for use under a CC0 license.

Supplementary Figure 9: ZNF207 zinc finger domain facilitates interactions with U1 snRNP components. Related to Figure 6.
(A) Schematic representation of ZNF207 variants.
(B) RT-PCR analysis of ACLY (top) and SRRM1 (bottom) alternative splicing in HEK293 cells treated with ZNF207-targeting siRNA and transiently transfected with siRNA-resistant, C-terminally 3×FLAG-tagged ZNF207 mutants. Quantification of ∆PSI values from three independent experiments are shown below the gel. Data are presented as mean ± SD. Statistical comparisons were performed relative to the siZNF207 sample without ZNF207 ORF expression. ****$p < 0.0001$; Dunnett's multiple comparisons test following one-way ANOVA.
(C) Western blot analysis of siRNA-resistant 3×FLAG C-terminal tagged ZNF207 mutants expressed in HEK293 Flp-In cells under doxycycline induction. Cells were treated with non-targeting siRNA control or siRNA targeting endogenous ZNF207 (siZNF207). Western blots were probed with antibodies against ZNF207, FLAG (to detect tagged constructs), and GAPDH (loading control).
(D) RT-PCR analysis of ACLY (top) and SRRM1 (bottom) alternative splicing in HEK293 Flp-In cells treated with ZNF207-targeting siRNA and/or expressing siRNA-resistant wild-type or mutant ZNF207 ORFs. Quantification of ∆PSI values from three independent experiments is shown below the gels. Data are presented as mean ± SD. Statistical comparisons were performed relative to the siZNF207 sample without ZNF207 ORF expression. ****$p < 0.0001$; Dunnett's multiple comparisons test following one-way ANOVA.
(E-F) Biotin-based RNA labeling of UV-crosslinked RNA bound to immunoprecipitated ZNF207 truncation mutants. Pull-down efficiency of each construct was assessed by FLAG Western blot. Quantification of relative RNA signal from three independent experiments is shown below the gel. Data are presented as mean ± SD. Statistical analysis was performed using one-way ANOVA followed by Dunnett's multiple comparisons test.
(G) Representative western blot analysis of total cell lysates (input) treated with benzonase, and FLAG immunoprecipitates (IP: FLAG-M2) from HEK293 Flp-In cells expressing 3×FLAG-tagged ZNF207 truncation mutants shown in S8C. Blots were probed with antibodies specific for FLAG, SNRPA, SNRNP70, RBM25, SF3B1, and GAPDH (negative control).
(H) Quantification of co-immunoprecipitated spliceosome components, normalized to FLAG-ZNF207 pulldown efficiency and wild-type (WT) ZNF207 levels. Data are presented as mean ± SD. Statistical comparisons were performed relative to WT-ZNF207. ***$p < 0.001$, **$p < 0.01$, *$p < 0.05$; Šídák's multiple comparisons test following two-way ANOVA.
.

bioRxiv preprint doi: https://doi.org/10.1101/2025.04.25.648738; this version posted April 27, 2025. The copyright holder for this preprint (which was not certified by peer review) is the author/funder. This article is a US Government work. It is not subject to copyright under 17 USC 105 and is also made available for use under a CC0 license.

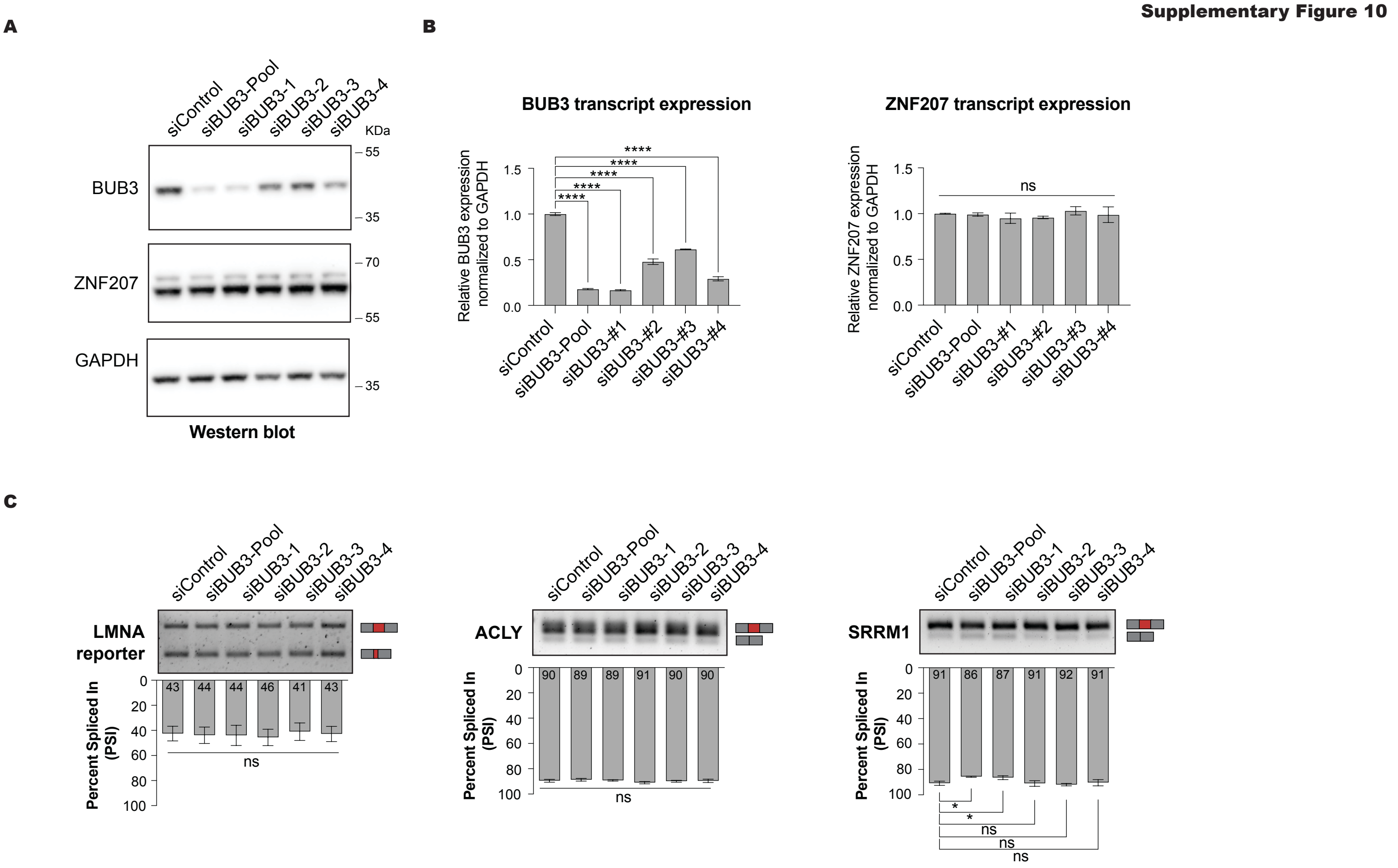

Supplementary Figure 10: BUB3 knockdown has minimal impact on ZNF207-driven splicing. Related to Discussion.
(A) Western blot analysis of BUB3 and ZNF207 in HEK293T cells treated with non-targeting siRNA control (siCTL), four independent siRNAs targeting BUB3 (siBUB3), or a pool of these siRNAs. Blots were probed with antibodies against BUB3, ZNF207, and GAPDH (loading control).
(B) Real-time quantitative RT-PCR analysis of BUB3 and ZNF207 transcript levels in HEK293T cells treated with either control siRNA (siCTL) or siRNAs targeting BUB3 (siBUB3), as in panel B. Quantification of transcript levels from three independent experiments are displayed. Data are presented as mean ± SD. ****p-value < 0.0001; one-way ANOVA with Dunnett's multiple comparisons test.
(C) RT-PCR analysis of *LMNA* minigene reporter (left), endogenous *ACLY* (middle), and endogenous *SRRM1* (right) alternative splicing in HEK293T cells treated with BUB3-targeting siRNAs, as in panel B. Quantification of PSI values from three independent experiments are shown below the gel. Data are presented as mean ± SD. *p-value < 0.05; one-way ANOVA with Dunnett's multiple comparisons test.
